# Impact of Natalizumab on Cognitive Performances and Fatigue in Relapsing Multiple Sclerosis: A Prospective, Open-Label, Two Years Observational Study

**DOI:** 10.1371/journal.pone.0035843

**Published:** 2012-04-25

**Authors:** Pietro Iaffaldano, Rosa Gemma Viterbo, Damiano Paolicelli, Guglielmo Lucchese, Emilio Portaccio, Benedetta Goretti, Vita Direnzo, Mariangela D'Onghia, Stefano Zoccolella, Maria Pia Amato, Maria Trojano

**Affiliations:** 1 Department of Neurosciences and Sense Organs, University of Bari Aldo Moro, Bari, Italy; 2 Department of Neurology, University of Florence, Florence, Italy; University Hospital of Heidelberg, Germany

## Abstract

**Background and Objectives:**

Natalizumab reduces the relapse rate and magnetic resonance imaging activity in patients with Relapsing-Remitting Multiple Sclerosis (RRMS). So far the influence of natalizumab on cognitive functions and fatigue in MS remains uncertain. The aim of this prospective, open-label, observational study was to evaluate the possible effects of natalizumab on cognition and fatigue measures in RRMS patients treated for up to two years.

**Methods:**

Cognitive performances were examined by the Rao's Brief Repeatable Battery (BRB), the Stroop test (ST) and the Cognitive Impairment Index (CII), every 12 months. Patients who failed in at least 3 tests of the BRB and the ST were classified as cognitively impaired (CI). Fatigue Severity Scale (FSS) was administered every 12 months to assess patient's self-reported fatigue. One hundred and 53 patients completed 1 and 2 year-natalizumab treatment, respectively.

**Results:**

After 1 year of treatment the percentage of CI patients decreased from 29% (29/100) at baseline to 19% (19/100) (*p* = 0.031) and the mean baseline values of CII (13.52±6.85) and FSS (4.01±1.63) scores were significantly reduced (10.48±7.12, *p*<0.0001 and 3.61±1.56, *p* = 0.008). These significant effects were confirmed in the subgroup of patients treated up to two years.

**Conclusions:**

These results demonstrate that a short-term NTZ treatment may significantly improve cognitive performances and fatigue in RRMS patients.

## Introduction

Cognitive Impairment (CI) and fatigue are common features of Multiple Sclerosis (MS), with an estimated prevalence ranging approximately from 40 to 65% of patients [1]–[4]. Information processing speed, abstract reasoning, executive functioning, sustained attention and long-term memory are the most common affected cognitive domains in MS [5]. CI detrimentally affects many aspects of MS patient' daily life, such as the ability to participate fully in society and to maintain employment with a consequent negative impact on the overall quality of life (QoL) [6]. In addition, the few data available on the natural history of CI and fatigue in MS showed they tend to worsen during the disease course [7]–[9] and correlate with disability progression [5], [10] measured by the Expanded Disability Status Scale (EDSS) [11]. Surprisingly, very little attention has been paid to cognition and fatigue related outcomes in randomized clinical trials (RCTs) of currently approved disease modifying drugs (DMDs), despite some data suggested their potential neuroprotective effects [12]–[14].

Improvement in measures of information processing speed, verbal memory and verbal learning has been reported in relapsing-remitting (RR) MS patients treated with intramuscular Interferon beta (IFNβ)-1a [15] compared with placebo. IFNβ-1b demonstrated to improve visual memory in RRMS [16] and information processing speed in patients with clinically isolated syndrome suggestive of MS [17], and to delay the cognitive decline in patients with secondary progressive MS [18]. Recently, a longitudinal observational study demonstrated a beneficial dose-related effect of subcutaneous IFNβ-1a on cognitive functions in RRMS patients [19]. Until now, the impact of the IFNβ on fatigue is still controversial [20]–[22].

Glatiramer Acetate (GA) treatment failed to demonstrate significant effects on cognition, even in a long-term follow-up [23], [24], whereas some significant effects of GA on fatigue have been shown [25].

A post hoc analysis of the two pivotal trials of natalizumab (NTZ), AFFIRM [26] and SENTINEL [27], suggested a positive effect of NTZ on both the physical and mental domains of Health Related QoL [28]. More recently, data from observational studies, involving small groups of patients [29], [30], or using only one cognitive test [31], suggested that NTZ treatment may impact cognitive performances and fatigue [32] in patients with RRMS.

Here we report the results of an open label, prospective, observational study aimed to further assess the effect of NTZ on cognition and fatigue in a large population of RRMS patients who were followed for up to two years.

## Methods

### Patients

All the MS patients who started the standard NTZ regimen (300 mg ev monthly) treatment in two Italian academic (Bari and Florence) MS centers from 2007 were followed through scheduled clinical and neuropsychological assessments. The relapses occurred in the last year prior to the treatment and every new relapse occurring during the treatment were recorded. Annualized relapse rate (ARR) in the last year pre-treatment (baseline) and at the end of the first and second year of treatment was calculated for each patient. A complete neurological examination including the EDSS score assessment was performed at baseline and every 3 months throughout treatment. Cognitive functioning, using the Rao's Brief Repeatable Battery (BRB) [5], [33] and the Stroop Test (ST), was measured before the first infusion and every 12 months by a trained psychologist in each center. BRB included tests of verbal memory acquisition and delayed recall (Selective Reminding Test – SRT), visual memory acquisition and delayed recall (10/36 Spatial Recall Test – SPART), attention, concentration and speed of information processing (PASAT 3; PASAT 2; Symbol Digit Modalities Test – SDMT), and verbal fluency on semantic stimulus (Word List Generation – WLG). Frontal lobe executive functions were assessed by the Stroop color-word task (ST). Versions A and B of the BRB were used alternatively at each examination.

Cognitive impairment was defined as the failure in at least 3 tests on BRB and ST using a 5^th^ percentile cut-off for each test, which corresponded to a z-score 2 standard deviation (SD) below the mean Italian normative values [33]. A global score, defined Cognitive Impairment Index (CII), allowing the evaluation of changes in cognitive performances independently by the number of cognitive tests failed at the BRB and the ST, was obtained using the mean and SD from the normative sample of Rao's battery and the ST [19], [34]. For each patient, a grading system was applied to individual cognitive tests, based on the number of SDs below the control mean (i.e. grade 0 was given if the patient scored at or above the control mean, 1 if he/she scored below the control mean, but at or above 1 SD below the control mean, and so on until all patient scores were accommodated). Finally, all the patient's scores were summed to give one overall measure of cognitive function.

Fatigue and depression were respectively, assessed, by the Fatigue Severity Scale (FSS) [35], using a cut-off value of 4.5 to identify patients with a level of fatigue interfering with daily activities, and by the Beck Depression Inventory (BDI) [36], using a cut-off value of 9 to identify patients with at least mild depressive symptoms, at baseline and every 12 months. If a relapse occurred at the time of scheduled neuropsychological assessments, cognitive testing was delayed until 30 days after the last steroid administration. Patients with a visual function impairment interfering with the performances in the cognitive tests were not included in the study. All the patients who reached 1 and 2 years of NTZ treatment entered into the analyses.

The local Ethics Committees of the University of Bari and of the University of Florence approved the study, and written informed consent was obtained from all patients prior to entering the study.

### Statistical analyses

Descriptive analyses were performed at the baseline. No imputation of missing data was considered. To test differences across different time points in related samples non parametric tests were used. The Wilcoxon signed – rank test for paired samples and the Friedman two-way test for repeated measures with post-hoc correction were used to compare mean values in paired examinations or when three or more evaluations were available for continuous variables. Cochran Q test for repeated measures and McNemar test for pairwise comparisons were used to assess changes over time in categorical variables. A value of *p*<0.05 was considered significant for the Wilcoxon signed – rank test, the Friedman two-way test and the Cochran Q test. For post-hoc multiple comparisons, we elected a pre-assigned *p*/number of comparisons (Bonferroni's correction) as the threshold for significance, and therefore, a *p*<0.017 (0.05/3) was used as a definition of statistical significance for n = 3 comparisons. Multivariate logistic regressions were performed to evaluate potential clinical and demographic confounders which can affect the changes of neuropsychological performances and fatigue scores at the end of the 1st year of NTZ treatment. In these models the following variables: sex, age, time spent in formal school education, disease duration, BDI score, time from the last steroid pulse to start of NTZ treatment, ARR in last year prior NTZ treatment, baseline EDSS, baseline FSS score and baseline CII, were included as covariates; “improved and stable" and “worsened" FSS score or CII were considered as dependent variables. Data analyses were performed by SPSS 17.0.

## Results

At the end of September 2011, 100 and 53 RRMS patients completed 1 and 2 years of treatment, respectively. Patients' demographic and clinical characteristics at baseline are shown in **Table 1**. In patients completing 1 year-NTZ treatment, the mean ARR at baseline (1.91±0.82) and the mean baseline EDSS score (3.66±1.14) significantly decreased at the end of follow-up (0.28±0.53, *p*<0.0001; 3.51±1.24, *p* = 0.005).

**Table 1:** Demographic and clinical characteristics at baseline.

Variable		100 patients	53 patients
		with 1 year	with 2 years
		NTZ	NTZ
		Treatment	Treatment
Sex (F/M)		72/28	37/16
Age at baseline (Mean ±SD)*		34.55±9.25	33.41±8.85
Disease duration in years (Mean ±SD)*		11.09±7.52	9.67±6.14
Educational level years (Mean ±SD)*		12.51±3.27	12.56±3.63
ARR (Mean ±SD)		1.91±0.82	1.98±0.87
EDSS (Mean ±SD)		3.66±1.14	3.58±1.12
Number of previous DMDs treatment (Median (Range))		2 (0–4)	2 (0–4)
Last DMD (n and %)	Interferon-beta	79 (79%)	42 (79.2%)
	Glatiramer Acetate	14 (14%)	7 (13.2%)
	Mitoxantrone	1 (1%)	0
	Azathioprine	2 (2%)	1 (1.9%)
	Treatment naïve	4 (4%)	3 (5.7%)
Previous treatment duration (Mean ±SD)†		53.99±41.53	55.99±41.67
BDI score (median; Range)		9 (0–32)	9 (0–28)
Absence/Presence of	No Depression (0–9)	56	28
Depression by the	Mild Depression (10–18)	26	13
BDI score	Moderate Depression (19–29)	17	12
	Severe Depression (30–63)	1	0

* Data expressed in years; †Data expressed in months

Abbreviations: NTZ = Natalizumab; ARR = Annualized Relapse Rate; EDSS = Expanded Disability Status Scale; DMD = Disease Modifying Drug; BDI = Beck Depression Inventory.

In the subgroup of patients receiving NTZ for two years, the mean ARR and the mean EDSS score significantly decreased during the treatment (Friedman test: *p*<0.0001 and *p* = 0.011, respectively). A Wilcoxon signed-rank test with a Bonferroni correction for multiple tests showed that the ARR significantly improved after 1 (0.23±0.42; *p*<0.0001) and 2 years (0.18±0.43; *p*<0.0001) in comparison to baseline value (1.98±0.87). There were no significant differences between year 1 and 2 of treatment (*p* = N.S.). A significant reduction of the baseline EDSS score (3.58±1.12) was also observed at 1 year (3.35±1.17, *p* = 0.001), whereas no significant difference was found at year 2 (3.48±1.43; *p* = N.S.). At the baseline the median BDI score was 9, ranging from 0 to 32. Forty-four patients had a BDI score higher than 9 and were classified as patients with depressive symptoms. (**Table 1**). The mean BDI score (10.36±6.94) significantly decreased after 1 (9.48±7.47; *p* = 0.001) and 2 years (n = 53; 9.07±6.74; *p* = 0.001) of NTZ treatment.

### Cognitive impairment

At baseline 29/100 (29%) of RRMS patients failed in at least three tests of the BRB and the ST and were classified as cognitively impaired. In patients completing 1 year-NTZ treatment, the number of patients cognitively impaired decreased to 19/100 (19%) (*p* = 0.031) (**Table 2**). Accordingly, the mean CII values were found to be significantly reduced at year 1 in comparison to baseline values (*p*<0.0001) (**Table 3**).

**Table 2:** Changes of proportion of RRMS patients with Cognitive Impairment (CI) after 1–2 year Natalizumab treatment.

Timepoint	Patients with CI	Patients without CI	Proportion of
			patients with CI
Baseline (n = 100)	29	71	29%
Year 1	19	81	19%
Baseline (n = 53)	12	41	22.6%
Year 1	10	43	18.9%
Year 2	9	44	17%

**Table 3:** Changes of mean Cognitive Impairment Index (CII) values and of the mean FSS Score over 1 and 2 years of NTZ treatment.

Parameter	Timepoint	Mean [SD]	*p* value
CII (n = 100)	Baseline	13.52 [6.85]	<.0001*
	Year 1	10.48 [7.12]	
CII (n = 53)	Baseline	12.94 [7.09]	<.0001†
	Year 1	9.64 [6.89]	<.0001§
	Year 2	8.26 [6.75]	.008θ
FSS (n = 100)	Baseline	4.01 [1.63]	.008*
	Year 1	3.61 [1.56]	
FSS (n = 53)	Baseline	4.30 [1.58]	.004 †
	Year 1	3.70 [1.41]	N.S.θ
	Year 2	3.49[1.75]	.001§

* Wilcoxon signed-rank test;

† Wilcoxon signed-rank test pair-wise comparison: Year 1 vs Baseline;

§ Wilcoxon signed-rank test pair-wise comparison: Year 2 vs Baseline;

θ Wilcoxon signed-rank test pair-wise comparison: Year 1 vs Year 2.

In the subgroup of patients receiving NTZ for two years, the percentage of cognitively impaired patients decreased from 22.6% (12/53) to 18.9% (10/53) at 1 year and to 17% (9/53) at 2 years, although this decrease did not achieve the statistical significance (Cochran Q test, *p* = N.S.)(**Table 2**).

In patients treated for up to 2 years the CII significantly decreased (Friedman test: *p*<0.0001). A Wilcoxon signed-rank test with a Bonferroni correction for multiple tests showed that the CII significantly improved after 1 (9.64±6.89; *p*<0.0001) and 2 years (8.26±6.75; *p*<0.0001) in comparison to baseline value (12.94±7.09). The CII also furtherly decreased in the second year of treatment in comparison to the year 1 value (*p* = 0.008) (**Table 3**).

The results of the multivariate logistic regression (**Table 4**) showed that educational school level positively impacted [OR:1.217; 95% Confidence Interval = 1.008–1.468; *p* = 0.041] the improvement of neuropsychological performances at the end of the 1^st^ year of treatment, whereas sex, age, disease duration, BDI score, time from the last steroid pulse to start of NTZ treatment, ARR in last year prior NTZ treatment, baseline EDSS, baseline FSS score did not have any influence.

**Table 4:** Baseline predictors of improvement of the Cognitive Impairment Index (CII) and of the Fatigue Severity Scale (FSS) at 1 year of NTZ treatment: multivariate logistic regression analysis.

Parameter	Baseline	OR	95%Confidence	*p* value
	Variable		Interval	
Improvement of CII	Constant	2.492		0.719
at year 1 (n = 100)	Sex	1.367	(0.399–4.688)	0.619
	Age	0.957	(0.894–1.025)	0.210
	Disease Duration	0.991	(0.913–1.077)	0.834
	**Educational School level**	**1.217**	**(1.008–1.468)**	**0.041**
	EDSS	0.984	(0.559–1.733)	0.956
	FSS score	0.844	(0.571–1.248)	0.396
	BDI score	1.021	(0.940–1.110)	0.621
	Time from the last steroid			
	Pulse	0.974	(0.760–1.250)	0.840
	ARR	0.736	(0.385–1.405)	0.352
Improvement of FSS	Constant	4.140		0.518
at year 1 (n = 100)	Sex	1.029	(0.317–2.865)	0.956
	Age	0.973	(0.917–1.032)	0.359
	Disease Duration	1.012	(0.940–1.090)	0.748
	Educational School level	0.966	(0.821–1.135)	0.672
	EDSS	0.848	(0.524–1.374)	0.504
	BDI score	1.028	(0.956–1.105)	0.460
	CII	0.974	(0.904–1.049)	0.487
	Time from the last steroid			
	Pulse	1.000	(0.820–1.220)	0.998
	ARR	0.948	(0.502–1.790)	0.869

Abbreviations: ARR = Annualized Relapse Rate; EDSS = Expanded Disability Status Scale; BDI = Beck Depression Inventory.

**Table 5** shows in detail the mean scores of each cognitive test at the different time points. A significant difference between the baseline values and those at 1 year was found only for SPART (*p* = 0.037), SDMT (*p*<0.0001) and PASAT 2 (*p* = 0.026) scores. In the subgroup of patients with two year treatment a significant difference in the SDMT (*p* = 0.001), PASAT 3 (*p* = 0.006) and PASAT 2 (*p*<0.0001) performances was still evident at the end of the follow-up in comparison to baseline values. The Wilcoxon signed-rank test with a Bonferroni correction for multiple tests showed that the SDMT, and the PASAT 2 performances significantly improved after 1 (*p* = 0.001 and *p* = 0.003, respectively) and 2 years (*p*<0.0001) in comparison to baseline values. There were no significant differences between year 1 and 2 of treatment (*p* = 0.088 and *p* = 0.080, respectively). Furthermore, in these patients an improvement in the PASAT 3 (*p* = 0.005) performances was found in the 2^nd^ year of treatment.

**Table 5:** Changes of mean scores for each cognitive test after 1 and 2 years of NTZ treatment.

	1 year Treatment (n = 100)		2 years Treatment (n = 53)		
	Baseline	Year 1	Baseline	Year 1	Year 2
**Verbal**					
**Memory**					
SRT-LTS	35.20 (13.90)	36.68 (13.58)	35.49 (14.10)	37.94 (13.94)	36.64 (15.09)
SRT-CTLR	26.83 (14.60)	26.67 (14.87)	28.15 (14.63)	28.32 (15.58)	28.24 (16.65)
SRT-D	7.11 (2.65)	7.54 (3.62)	7.13 (2.55)	7.98 (4.41)	7.34 (3.93)
**Spatial**					
**Memory**					
SPART	17.65 (6.14)	18.90 (6.04)*	18.58 (6.36)	18.40 (6.47)	19.68 (5.99)
SPART-D	6.30 (3.48)	6.51 (2.55)	6.85 (4.10)	6.66 (2.56)	6.60 (2.17)
**Attention**					
SDMT	42.87 (12.33)	46.29 (12.44)**	43.32 (12.99)	48.06 (12.09)	49.57 (12.92)†
PASAT-3	34.01 (14.02)	34.24 (14.94)	35.75 (14.53)	36.34 (14.80)	38.88 (14.46)†
PASAT-2	25.61 (12.81)	28.09 (12.85)*	26.94 (14.25)	31.03 (13.12)	32.45 (13.59)†
**Executive**					
**Function**					
WLG	19.98 (6.02)	20.48 (5.79)	20.81 (5.66)	20.41 (5.42)	22.15 (5.48)
ST	70.90 (15.18)	69.98 (18.45)	69.35 (13.70)	69.40 (13.27)	65.42 (14.30)

* p<0.05 (Wilcoxon signed-rank test);

** p<0.0001 (Wilcoxon signed-rank test);

† p<0.01 (Friedman two-way test);

Abbreviations: SRT-LTS = Selective Reminding Test – Long-Term Storage; SRT-CTLR = Selective Reminding Test – Consistent Long-Term Retrieval; SRT-D = Selective Reminding Test –Delayed; SPART = 10/36 Spatial Recall Test; SPART-D = 10/36 Spatial Recall Test-Delayed; SDMT = Symbol Digit Modalities Test; PASAT 3 = Paced Auditory Serial Addition Test three second rate; PASAT 2 = Paced Auditory Serial Addition Test two second rate; WLG = Word List Generation; ST = Stroop Test.

Values are expressed as Mean (SD).

### Fatigue

At baseline the mean FSS score was 4.01 (±1.63), and 45/100 patients (45%) reported levels of fatigue which interfered with daily activities (FSS score ≥4.5). After 1 year of NTZ treatment, the mean FSS score significantly decreased to 3.61 (±1.56) (*p* = 0.008) (**Table 3**) and the proportion of patients with FSS score ≥4.5 decreased to 29% (29/71, *p* = 0.005). In patients with a 2 year-follow-up, the mean FSS score significantly decreased during the treatment (Friedman test, *p* = 0.001). A Wilcoxon signed-rank test with a Bonferroni correction for multiple tests showed that the mean FSS score significantly improved after 1 (*p* = 0.004) and 2 years (*p* = 0.001) in comparison to baseline values. There were no significant differences between year 1 and 2 of treatment (*p* = N.S.) (**Table 3**). In this subgroup the proportion of patients with FSS score ≥4.5 significantly (Cochran Q test, *p* = 0.01) decreased during the 2 years NTZ treatment from 52.8% (28/53 patients) to 32.1% (17/53 patients) at year 1 and to 34% (18/53 patients) at year 2. A McNemar test with a Bonferroni correction for multiple tests showed a trend toward the reduction of the proportion of patients with FSS score ≥4.5 after 1 (*p* = 0.019) and 2 years (*p* = 0.021) in comparison to baseline value. There were no significant differences between year 1 and 2 of treatment (*p* = N.S.).

The results of the multivariate logistic regression (**Table 4**) did not show any significant effect of the baseline covariates (sex, age, time spent in formal school education, disease duration, BDI score, time from the last steroid pulse to start of NTZ treatment, ARR in last year prior NTZ treatment, baseline EDSS, and baseline CII) on the improvement of FSS score at the end of the 1^st^ year of treatment

## Discussion

The results of this observational study confirm the efficacy of NTZ treatment in reducing disease activity and disability progression in RRMS patients already demonstrated in NTZ pivotal trials [26], [27]. In addition they show that a short-term NTZ treatment may significantly improve cognitive performances and fatigue in these patients. A decrease of the proportion of cognitively impaired MS patients and an improvement of sustained attention, information processing speed and visuo-spatial memory, as measured by the SDMT, PASAT 2 and SPART, were evident since the first year of NTZ treatment. Most of these beneficial effects on cognition were still present after two years of treatment. These findings are consistent with previously published Italian [29], [30] and Swedish [31] observational studies suggesting a beneficial effect of NTZ on cognitive performances. In the Italian study, even if a smaller number of patients (n = 39 at 1 year; n = 11 at 2 years) than in the current study was evaluated and the cognitive impairment was assessed by a neuropsychological battery different from the BRB we used, an improvement in tests which explore information processing speed, executive function and memory tests were also found [29], [30]. Data from the Swedish post-marketing surveillance program of MS patients treated with NTZ for up to 2 years, clearly demonstrate an improvement in SDMT [31].

It is noteworthy that in the current study we used, also, a measure of the global change of cognitive performances that is the CII. We found a significant improvement in the mean CII value after 1 year NTZ treatment and this value was furtherly improved in the second year of treatment. The CII [19], [34] may be considered a more suitable tool to evaluate changes in cognitive performances than considering the individual tests, because CII is independent by the number of cognitive tests failed at the BRB and the ST (i.e. if an individual patient was impaired in six tests at baseline and failed four tests at follow-up, that patient would be still considered cognitively impaired, despite the fact that his or her performances were objectively improved), therefore its use strengthens the results of this study. Moreover in this study we used alternate versions of cognitive tests, and adequate time intervals between them to reduce the major methodological issue when neuropsychological tests are repeated over time that is the practice effect [37], [38].

The significant improvement of fatigue measures after 1 and 2 years of NTZ treatment that we have found, is also in line with a previous German study [32] that showed a decrease of fatigue in RRMS treated with NTZ for 6 months.

The effect of potential clinical and demographic confounders which could affect the changes of neuropsychological performances and fatigue scores at the end of the 1st year of treatment was ruled out by a logistic regression analysis that demonstrated that both cognitive impairment and fatigue were independent from other clinical variables, in this cohort. The only covariate with a positive predictive effect on the neuropsychological performances was the educational level, as measured by the time spent in formal school education, in accordance with recent reports [39]–[41] showing a significant beneficial effect of the so-called brain reserve on cognition and on the brain atrophy progression in MS.

The degree and the time-trend of the improvement induced by NTZ on cognitive functions and fatigue in the current and previous studies [29]–[32] seem to suggest that these two phenomena could be, at least in mild disabled patients, more related to inflammation than to neurodegeneration mechanisms. A growing evidence seems to support this view. In the experimental autoimmune encephalomyelitis (EAE), the rodent model of MS, it was recently demonstrated that the activation of microglia and the inflammatory cytokines released from infiltrating lymphocytes, especially TNF-α, are able to alter synaptic transmission [42], [43]. This induced synaptopathy is related to cognitive dysfunction in this experimental model of MS [42], [43]. The significant reduction of cerebrospinal fluid (CSF) and plasma levels of pro-inflammatory cytokines and of CSF levels of the light-chain neurofilament, a marker of axonal loss, during the NTZ treatment [14], [44], [45] may be responsible of the cognitive dysfunction improvement induced by NTZ. Moreover 1 year NTZ treatment has been demonstrated to prevent the accumulation of active cortical lesions in RRMS, an MRI measure that is correlated with cognitive decline [46].

We recognize that the main limit of this observational study is the lack of a concurrent control group. However it must be considered that many barriers may prevent the execution of a controlled observational study aimed to evaluate the effects of a second line drug (i.e. NTZ) on a population of MS patients with high disease activity. The inclusion of a placebo group, that remains untreated for up to 2 years, is not feasible and ethical. Moreover a comparison between patients treated with a second line treatment (NTZ) and patients treated with a first line treatment (IFN β or GA) is also difficult to carry out, because the unbalanced baseline measures of disease activity (i.e. ARR, EDSS, MRI) between the two groups (indication bias) may influence the measured association between exposure and outcome. Within-subject methods, including self-controlled case-series method [47] as we used in this study, in which outcomes are compared between periods before and after treatment exposure within the same individuals, are less susceptible to confounding by indication and may eliminate between-person confounding [48].

Nevertheless an analysis is ongoing aimed to identify a suitable active drug control group among the DMD treated MS patients collected, in the same period of time, in the MS databases (Imed-web) in Bari and Florence Centers. We are using a propensity score matching technique [49], [50], which is the most common method currently used to reduce bias in treatment comparisons in observational studies [51], [52] to select patients whose demographics and disease characteristics overlap with those of our cohort of NTZ-treated patients, and that were evaluated for cognitive impairment at the same time-point of the NTZ-treated cohort. A preliminary analysis [data not shown] on a sample of 20 RRMS patients (mean ± SD: age: 35.12±10.99; disease duration: 10.32±9.92; mean EDSS 3.5±1.14; mean ARR in the year before the treatment initiation: 1.95±0.85) treated with different formulations of DMDs was performed. The results showed that DMD treatment reduced the baseline mean CII values over a two year follow-up (mean [SD] Baseline CII: 14.61 [6.62]; mean [SD] 1 Year CII: 13.06 [6.03]; mean [SD] 2 Years CII: 12.78 [6.06] in these sample of patients, but this effect did not reach a statistical significance, Friedmann two-way test: p = 0.241). However the lack of statistical significance could be due to the small sample size of this DMD treated group. So far, few studies reported some beneficial effects of IFN beta treatment on cognitive functions in RRMS patients [15]–[19], whereas GA treatment failed to demonstrate any significant effect on cognition [23], [24], but only some significant effects on fatigue measures [25].

Although no direct comparison could be made, our findings allow to speculate that the effects of NTZ on both cognition and fatigue measures might be greater than first line DMDs.

A longer follow-up in a larger population, including a propensity score matched control group, is needed to confirm whether the early beneficial effects of NTZ in improving cognitive performances and fatigue, we found in this study, hold-up in the long-term.
